# Grand Challenges in Nutrition and Food Science Technology

**DOI:** 10.3389/fnut.2014.00004

**Published:** 2014-04-07

**Authors:** Chor San Khoo, Dietrich Knorr

**Affiliations:** ^1^International Life Sciences Institute North America, Washington, DC, USA; ^2^Technische Universität Berlin, Berlin, Germany

**Keywords:** nutrition security, food and health, food regulation, product innovation, food science technology

## Grand Challenges

The twenty-first century is marked by multiple unprecedented environmental challenges that could threaten human survival. The combined impact of climate change, energy and water shortage, environment pollutants, shifting global population demographics, food safety, and growing disease pandemics, all place undue stress on the planet’s food system, already in a sensitive balance with its ecosystem. These threats, natural or man-made, obligate the scientific community to proactively seek new breakthrough food and nutrition solutions to insure global food sustainability and nutrition security in the future. To achieve this, innovative solutions need to be considered throughout the whole food chain inclusive of food choices and dietary patterns in order to make any significant improvements in the food supply, nutritional, and health status.

Any changes to the food supply will inevitably impact food, nutrition, and health policies, particularly pertaining to food production, agricultural practices, dietary patterns, nutrition, and health guidance and management. Climate change, droughts, and floods have exacerbated concerns related to land availability for agriculture usage, animal and crop production, as well as quality and yield. The resulting effect of energy shortage has directed attention to bio fuel as an alternate source of energy, particularly from crops such as corn and oil seeds. This sets the stage for an added competitive demand for crops being used as fuel in addition to food and feed. As a result, there is an urgent need to find alternative solutions to improve the efficiency and sustainability in the food supply chain by reducing food waste and enhancing nutritional qualities of foods through fortification technologies (i.e., biofortification and chemical fortification).

Another area receiving increasing research interest is food safety. The presence of chemical contaminants in the food chain, such as PCB and dieldrin, is particularly troubling. In addition, the emergence of new food pathogens, particularly viruses, as well as the re-emergence of known food pathogens, have captured considerable research attention. For example, alteration to diet pattern and nutrition affect microbial population, with consequential effect on the immune system, disease pathogenesis and health (1, 2). To elucidate microbiome interactions with food, nutrients, and food substances, and their potential involvement in disease ecology, novel approaches adapted from various disciplines are needed.

The United Nations projected that by 2050 the world population would reach 9.6 billion (3). The 60+ adult population will constitute 19% (2 billion) and 27% (3 billion) of world population by 2050 and 2100, respectively. There will be proportionally more women than men in the 60 and 80+ age groups by 2050 (4). Concomitant with the aging trend is the increase in the number of older adults with mental and physical disabilities (5). These facts necessitate new solutions to address primary and secondary prevention care and geriatric research. For the first time, the major cause of global deaths (36 million or 63%) will be from non-communicable diseases (NCD) instead of infections. Four categories of NCDs account for 80% (20 million) of global mortality causes: cardiovascular disease, cancer, diabetes, and chronic respiratory diseases (6). The World Health Organization estimates that over 20 million deaths can be prevented by reducing the exposure level to key modifier risks such as unhealthy diets, physical inactivity, tobacco, and alcohol use (7). Thus far, obesity, hypertension, cardiovascular diseases, and diabetes continue to pose significant risks at pandemic proportion. Of further concern is the growing number of children who are overweight and at risk of obesity, sedentary behavior, and early onset of type 2 diabetes. Taken together, obesity, sedentary behavior, diet-related diseases, such as cancer, cardiovascular diseases, strokes, mental health problems, chronic malnutrition, and maternal-infant health represent areas of growing concern and research challenges.

### Stalemate in health-policy implementation

To address the emerging food and health issues, countries have issued national food, nutrition, and health guidelines and regulations. Unfortunately, in spite of this, many of these food and nutrition guideline goals remain unmet. An example is the United States Dietary Guidelines for Americans, issued first in 1980. Since then, seven Dietary Guidelines (DGs) have been issued; the latest released in 2010 (8). The DGs contain dietary recommendations for Americans for balanced nutrition and optimal health. They include, for example, recommendations to reduce the intake of dietary sodium, cholesterol, saturated fats, and sugars, while increasing the consumption of whole grains, vegetables, and fruits, as well as low fat dairy products. Yet after 30 years, recommendations for sodium, saturated fats, vegetables, fruits, and whole grains remain unmet.

A reduction in sodium intake remains particularly challenging due to the versatile role sodium chloride plays in food functionality, palatability, and health. The basic understanding of salt taste mechanisms and the physiology of sodium homeostasis in humans continues to be an active area of research and has impeded advances in the development of optimal and versatile salt reduction technology tools for food products. On the other hand, efforts to reduce fat and cholesterol intake have been more successful, with technologies having advanced sufficiently to develop products that meet both consumer taste and value expectations. However, this process took more than 20 years!

Important lessons learned from these findings: guideline recommendations that precede food technology capability will face considerable challenges of meeting set goals; even if a technology is available, time and research investments are needed to apply the technology to multiple food systems and categories; the need to understand basic mechanisms of food substance interactions is important to enable specific and widespread applications of relevant technologies; consumer acceptance of foods is critical to the success of any set of recommendation guidelines; sufficient time is needed to allow consumers to change their food and dietary behavior; multidisciplinary experts from all sectors, with research and application experience in food science and technology, nutrition, medicine, consumer behavior, economics, etc., should be part of the recommendation deliberation to ensure implementation and long term compliance and success.

### Need for research investment and scientific integration

In 2011, the Foresight Report (9) concluded that “Investment in research on modern technologies is essential in light of the magnitude of the challenges for food security in the coming decades.” The complexity of the issues necessitates the development, application, coordination and sharing of integrated research approaches, and solutions across disciplines. Over the past decades, many different technologies and methodologies have been adapted from various disciplines that are now being applied to food and nutrition research. These technologies include nanotechnology, bioinformatics, genetics, chemical and biofortification, -omics, decision-analysis science, big-data analyses, risk assessment and management frameworks, neuroscience, optics, photonics, and neuroimaging.

### Setting focus and priority areas

The scope of the challenges is daunting and the path forward can be confusing in absence of a clear focus and set priorities. In 2010, the Institute of Food Technology (10) narrowed the focus in health and wellness to personalized nutrition, molecular biology, and microbial ecology. The American Society for Nutrition (11) and others (12) identified several priorities for nutrition research: individual variability in response to diet and foods; early nutrition needs for reproduction, growth, development; nutrition in health maintenance; nutrition in medical management; understanding nutrition related behaviors; understanding the food supply/environment. There are also more specific research areas to target including variability of living systems (e.g., microbiome, biological networks, tissue specificity); optimal function and energy balance; disease progression and care for sub populations; drivers of food choices; nutrition and brain functions and imprinting; drivers of food choices; composition of novel ingredients. And finally, there are suggestions to consider cross-disciplinary tools/approaches such as -omics, bioinformatics, databases, biomarkers, and cost-effective analyses.

Perhaps the most comprehensive path to building a future framework template is best summed up in the European Technology Platform report “Food for Life Strategic Research and Innovation Agenda” (9). The report reflects the European 2020 and beyond vision, which provides an integrated role of food science, nutrition, and consumer sciences. From this vision, three key challenges emerge: improved health, wellbeing, and longevity (“Life to years”); consumer trust in the food chain; sustainability. To gain consumer confidence in food products and the food system, powerful concepts for high-quality products with new properties/functionalities have been developed. These concepts aim at achieving preference, acceptance, and fulfilling the needs of the consumers via gentle processes, including, for example, the PAN concept and the reverse engineering concept. Another important aim is to describe the physiological impact of changes in food structures in the context of process–structure–functional property relationships in order to prevent deleterious changes, which negatively affect product quality, nutrient bioavailability, and functionality (www.fooddrinkeurope.eu).

### Framework for meeting the grand challenges in nutrition and food science technology

Based on these findings and considering the grand challenges related to food and health, future developments in nutrition, food science, and technology need to focus on the following points:

Food, nutrition, and health
  - role of food and nutrition throughout life cycle in support of optimal health and improvement of quality of life
  - maternal-child and geriatric nutrition
  - role of food and nutrition in disease etiology and management
  - understanding individual variability in response to food and system biology variability and implications for setting guidelines
  - advancing nutrigenomics and addressing personalized nutrition for optimal health.
Food system and technology
  - food and water security and safety
  - energy management of foods (fighting hunger as well as obesity)
  - increasing sustainability within the food chain including food loss and food waste reduction
  - addressing challenges of climate change
  - providing food concepts for an aging population and for personalized nutrition (or at least for specific target groups)
  - regaining consumer trust in the food supply
  - improving existing and providing new functions/properties of foods via targeted processing (“tailor-made foods”)
  - developing better tools for process control and development
  - integration of the food chain
  - involving food science and technology experts from all sectors at early discussion stages and deliberations on food, nutrition, and health guidelines
  These challenges for nutrition and food science technology can only be met through integrating disciplines, and through active interactions, collaborations, and partnerships.

## Conflict of Interest Statement

The authors declare that the research was conducted in the absence of any commercial or financial relationships that could be construed as a potential conflict of interest.
